# Adjunctive Use of Phage Sb-1 in Antibiotics Enhances Inhibitory Biofilm Growth Activity versus Rifampin-Resistant *Staphylococcus aureus* Strains

**DOI:** 10.3390/antibiotics9110749

**Published:** 2020-10-29

**Authors:** Lei Wang, Tamta Tkhilaishvili, Andrej Trampuz

**Affiliations:** 1 Center for Musculoskeletal Surgery, Charité—Universitätsmedizin Berlin, Corporate Member of Freie Universität Berlin, Humboldt-Universität zu Berlin and Berlin Institute of Health, 13353 Berlin, Germany; lei.wang@charite.de (L.W.); tamta.tkhilaishvili@charite.de (T.T.); 2 Berlin-Brandenburg Center for Regenerative Therapies, Charité—Universitätsmedizin Berlin, 13353 Berlin, Germany

**Keywords:** rifampin-resistant *Staphylococcus aureus*, biofilm infection, antibiotic-phage combination, synergism, suppression therapy

## Abstract

Effective antimicrobials are crucial for managing *Staphylococcus aureus* implant-associated bone infections (IABIs), particularly for infections due to rifampin-resistant *S. aureus* (RRSA). Failure to remove the implant results in persistent infection; thus, prolonged suppressive antibiotic therapy may be a reasonable alternative. However, a high incidence of adverse events can necessitate the discontinuation of therapy. In this scenario, commercial *Staphylococcal* bacteriophage Sb-1 combined with antibiotics is an option, showing a promising synergistic activity to facilitate the treatment of biofilm infections. Therefore, we evaluated the efficacy of the inhibitory activity of five antibiotics (doxycycline, levofloxacin, clindamycin, linezolid, and rifampin) alone or combined with phage Sb-1 (10^6^ PFU/mL) in a simultaneous and staggered manner, to combat five clinical RRSA strains and the laboratory strain MRSA ATCC 43300 in 72 h by isothermal microcalorimetry. The synergistic effects were observed when phage Sb-1 (10^6^ PFU/mL) combined with antibiotics had at least 2 log-reduction lower concentrations, represented by a fractional biofilm inhibitory concentration (FBIC) of <0.25. Among the antibiotics that we tested, the synergistic effect of all six strains was achieved in phage/doxycycline and phage/linezolid combinations in a staggered manner, whereas a distinctly noticeable improvement in inhibitory activity was observed in the phage/doxycycline combination with a low concentration of doxycycline. Moreover, phage/levofloxacin and phage/clindamycin combinations also showed a synergistic inhibitory effect against five strains and four strains, respectively. Interestingly, the synergistic inhibitory activity was also observed in the doxycycline-resistant and levofloxacin-resistant profile strains. However, no inhibitory activity was observed for all of the combinations in a simultaneous manner, as well as for the phage/rifampin combination in a staggered manner. These results have implications for alternative, combined, and prolonged suppressive antimicrobial treatment approaches.

## 1. Introduction

*Staphylococcus aureus* implant-associated bone infections (IABIs) represent a substantial challenge in orthopedic and trauma-related surgery, with severe consequences for the patients, such as long hospital stays and repeated surgeries [1]. Biofilm formation on the surface of the implant, in particular, plays a primary role in the initial development and chronic progression and/or recurrence of an infection [2]. Different pre-clinical studies revealed that the minimum inhibitory concentration for adherent bacterial cells are much higher (approximately 1000 times) than their planktonic counterparts, and it is impossible to reach by conventional antibiotic therapy due to the toxicities of antibiotics and the limitation of renal and hepatic functions [3].

Current treatment approaches for *S. aureus* implant-associated infections are based on surgical debridement with retention or removal of the infected tissue in order to reduce the bacterial burden as much as possible and on prolonged administration of biofilm-active antibiotics; the recommended antimicrobials are rifampin-based combination therapy due to its excellent bone penetration ability and oral bioavailability [4,5]. However, rifampin resistance emerged, frequently observed with the inappropriate use of rifampin [6]. Interestingly, several studies showed that the sub-inhibitory concentrations of fluoroquinolones induce the emergence of rifampin-resistance mutants [7,8,9]. Indeed, the rifampin-resistant *S. aureus* (RRSA) IABIs are considered as difficult to eradicate in patients with limiting therapeutic interventions. A report demonstrated that the implant survival rate applying antibiotic suppressive therapy with a five-year infection-free period was 20% percent higher than the non-suppression group [10]. Therefore, antibiotic suppressive therapy, as the alternative treatment strategy, is used to achieve sustained remission in chronic IABIs patients retaining the foreign body, as due to various reasons they are unable to remove the implant with the rifampin-resistant *S. aureus* infections.

Despite the emergence of RRSA IABIs challenging the use of recommended antimicrobials, a successful and efficient suppressive therapy option of current antibiotics is scarce. Several studies showed that the successful rates of antibiotic suppressive therapy range from 8 to 86% [10,11]; thereby, the necessity to pursue other alternative antimicrobial strategies, such as phage therapy, are taken into consideration. *Staphylococcal* phage Sb-1 is fully sequenced and a strictly lytic phage, it does not contain any bacterial virulence-associated genomes, making it acceptable for antimicrobial therapy [12]. Previous studies highlighted that *Staphylococcal* phage Sb-1 could disrupt the extracellular polymeric substances (EPS) of biofilm and facilitate and target the deep layer of the *S. aureus* cells, resulting in cell death and release of sub-particles [13]. It has been demonstrated that the phage–antibiotic combination therapy maybe more effective than unilateral treatment [14,15]. However, little is known about the inhibitory biofilm growth activity of the phage–antibiotic combination against *S. aureus* biofilm. In this context, the implementation of prolonged phage-antibiotic suppressive therapy has potential safety in the attempt to reduce the side effects of conventional antibiotics and to prevent the relapse of rifampin-resistant *S. aureus* IABIs without removal of the implants and avoiding disabling surgeries for patients not eligible for revision surgery.

Inhibitory biofilm growth activity cannot be detected by routine susceptibility testing, and numerous studies showed that isothermal microcalorimetry (IMC) is a reliable and nondestructive assay to detect microbial viability in terms of precise real-time monitoring of the heat produced due to the microbial metabolism [16,17,18,19]. Therefore, we used IMC to monitor the inhibitory biofilm growth activity with rich medium incubation for 72 h at 37 °C in the absence of antimicrobial agents. To the best of our knowledge, studies reporting the inhibitory activity of the efficacy of phage Sb-1 and antibiotic combinations against RRSA biofilms are scarce or limited. In the present study, the aim was to evaluate the efficacy of the inhibitory biofilm growth activity of different classes of antibiotics (doxycycline, levofloxacin, clindamycin, linezolid, and rifampin) alone or in association with *Staphylococcal* phage Sb-1, used as suppressive therapy, by either a simultaneous or staggered application in IMC for 72 h, to combat five clinical strains of rifampin-resistant, biofilm-producing *S. aureus* and the reference laboratory strain MRSA ATCC 43300.

## 2. Method

### 2.1. Bacteria and Bacteriophage

Five clinical strains of rifampin-resistance *Staphylococcus aureus* (RRSA) (listed in Table 1), including three methicillin-susceptible *S. aureus* and two methicillin-resistant *S. aureus* strains, were collected between 2015 and 2019. The laboratory strain MRSA ATCC 43300 was used as a reference strain. A broth microdilution assay was performed to confirm that the minimum inhibitory concentrations (MICs) of rifampin were greater than or equal to 1 μg/mL for the clinical strains according to EUCAST breakpoints [20], which was interpreted as rifampin-resistance. The phage Sb-1 infecting all the strains was supplied from the Georgia Eliava Institute (Tbilisi, Georgia).

### 2.2. Antimicrobial Agents and Susceptibility Testing

Doxycycline injectable solution (5 mL; Ratiopharm, Heppenheim, Germany), levofloxacin injectable solution (100 mL, Fresenius Kabi, Bad Homburg, Germany), linezolid injectable solution (Pfizer Pharma, Berlin, Germany), clindamycin powder (600 mg, Chephasaar, Ingbert, Germany), and rifampin powder (Sandoz Pharmaceuticals, Steinhausen, Switzerland) were purchased from the respective manufacturers. The MICs indicating various antimicrobial susceptibility were determined by the broth microdilution assay (BMD) in a brain heart infusion (BHI) [21]. An inoculum of approximately 5 × 10^5^ CFU/mL was used. Two-fold serial dilution of each antimicrobial agent was prepared in a 1 mL medium with plastic tubes and incubated for 24 h at 37 °C. The MIC for each antibiotic was defined as the lowest concentration of antimicrobial agent that completely inhibited the visible growth of the planktonic form. All experiments were performed in triplicates.

The susceptible strains were tested by a plaque assay in order to obtain the efficacy of the plating (EOP) for phage Sb-1 [13]; the EOP value was calculated as the ratio between the plaque forming units (PFU) on target clinical strains and MRSA ATCC 43300, which was used as the reference strain. All experiments were performed in triplicates.

### 2.3. Evaluation of the Synergistic Phage–Antibiotics Combinations Inhibiting Biofilm Growth Activity by IMC

The inhibitory biofilm growth activity of the antimicrobials against the *S. aureus* strains was determined by isothermal microcalorimetry (IMC), as described previously [13,17,22]. Briefly, a 24 h-old *S. aureus* biofilm was formed on porous glass beads at 37 °C; subsequently, the mature *S. aureus* biofilms were washed three times with sterile 0.9% NaCl to remove the planktonic form, and then were exposed to serial two-fold dilutions of the antimicrobials in 1 mL of BHI for a further 24 h incubation at 37 °C. After the 24 h exposure, the glass beads were rinsed (with 0.9% saline, and then directly inserted into the microcalorimetry ampoules with 3 mL of fresh BHI medium for the assessment of biofilm inhibition. IMC was applied in our study to evaluate the effect of antimicrobials on bacterial growth by monitoring the impact caused on the heat-flow curve, such as a reduction and/or delay in heat production compared to the growth control. According to this, the minimum biofilm inhibitory concentration (MBIC) was defined as the lowest concentration of antimicrobial agent that strongly inhibited biofilm metabolism and led to the absence of heat-flow production after 72 h of incubation at 37 °C. The *S. aureus* biofilms with BHI medium and only BHI medium were referred to as the growth control and negative control, respectively. Measurements were performed in triplicate. Moreover, the synergistic effect of the combined treatment (phage + antibiotics) against the rifampin-resistant *S. aureus* biofilms was evaluated in the simultaneous or staggered manner; the biofilms were formed and washed as described above. Finally, a 10^6^ PFU/mL titer of phage Sb-1 were used for all the experiments. Briefly, for the simultaneous exposure, 24 h-old biofilms were exposed to 1 mL of fresh BHI media containing the combination of phage Sb-1 (10^6^ PFU/mL) and serial dilutions of antibiotics for a further 24 h at 37 °C. In the staggered exposure, 24 h-old biofilms were firstly exposed to 1 mL phage Sb-1 (10^6^ PFU/mL) for 24 h and then to a serial dilution of antibiotics for a further 24 h. After application, the MBIC of the phage–antibiotic combination was assessed by microcalorimetry, as described above. The *S. aureus* biofilms with BHI medium were referred to as the growth control and sterile beads served as the negative control.

The interactions of the phage–antibiotic combinations were assessed by the fractional biofilm inhibitory concentration (FBIC) formula as described in previous studies [13,16], with some modification. The FBIC was defined based on the MBICs of the individual antibiotic inhibitory biofilm growth activity in the presence of bacteriophages: FBIC = MBIC_phage_/MBIC_alone_, where MBIC_phage_ corresponds to the obtained MBIC value of the individual antimicrobial tested in combination with the phage and the MBIC_alone_ represents the obtained MBIC value of the same antibiotic when tested alone. Synergism was defined as an FBIC ≤ 0.25, which correlated with a reduction of more than 2 × MBIC_alone_. If the FBIC > 0.25, there is no synergistic activity.

## 3. Results

### 3.1. The Bacterial Susceptibility to Antibiotics and Sb-1

**Figure 1:**
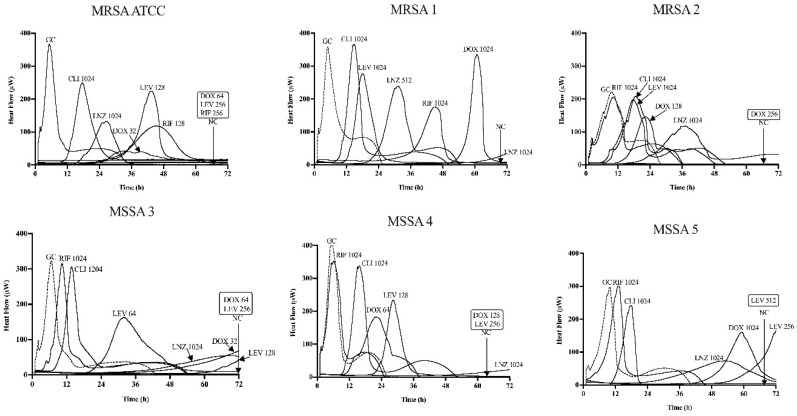
Microcalorimetry analysis of MRSA ATCC 43300 and five rifampin-resistance *S. aureus* biofilms inhibited by single antibiotics at different concentrations. Each curve shows the heat produced by viable bacteria presented in the biofilm after 24 h of antibiotic application. Numbers represent concentrations of the antibiotics (in μg/mL) doxycycline (DOX), levofloxacin (LEV), linezolid (LNZ), clindamycin (CLI), and rifampin (RIF). Circled values represent the MBIC, defined as the lowest antimicrobial concentration leading to biofilm metabolism inhibition in the absence of antimicrobials for 72 h. GC, growth control; NC, negative control. Data of a representative experiment are reported.

**Figure 2:**
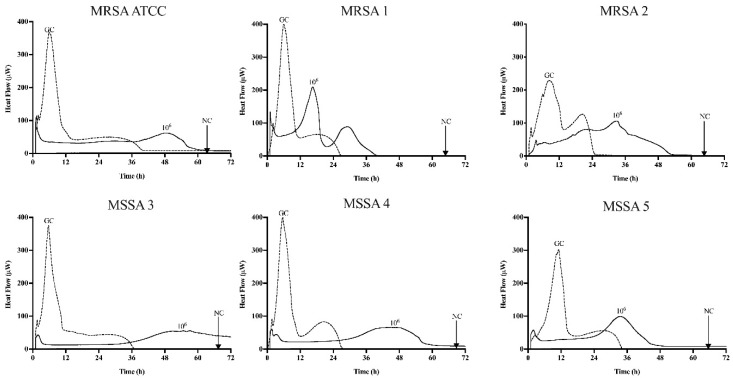
Microcalorimetry analysis of MRSA ATCC 43300 and five rifampin-resistance *S. aureus* biofilms inhibited by Sb-1. Each curve shows the heat produced by viable bacteria presented in the biofilm after 24 h of exposure to Sb-1. Numbers represent concentrations of Sb-1 (in PFU/mL). GC, growth control; NC, negative control. Data of a representative experiment are reported.

Table 1 summarize the susceptibility of planktonic (MIC) and the minimum biofilm inhibitory concentration (MBIC) for the MRSA ATCC 43300 and five rifampin-resistant *S. aureus* strains by BMD and by IMC, respectively. Additionally, Figure 1 and Figure 2 show the MBIC values of the antibiotics and phage Sb-1 in IMC, respectively.

Most strains exhibited susceptibility to the tested antibiotics according to EUCAST breakpoints (EUCAST, 2020), while doxycycline (2 µg/mL), levofloxacin (1 µg/mL), linezolid (4 µg/mL), clindamycin (0.5 µg/mL), and rifampin (0.5 µg/mL) were considered resistant. Doxycycline and levofloxacin displayed large variations in six strains of MICs, and two of the six (33%) presented as doxycycline-resistant and levofloxacin-resistant strains, respectively. While all of the six strains showed susceptibility to linezolid and clindamycin with small variation, 1~2 μg/mL and 0.25~0.5 μg/mL, respectively, the MRSA ATCC was resistant only to clindamycin. Additionally, rifampin showed superior resistance to all of the five RRSA strains, except for the reference strain. Based on the results of the spot assay performed with Sb-1 serial dilutions, all of the test strains were susceptible to Sb-1, and it showed a high EOP, ranging from 0.5 to 0.9, indicating that Sb-1 was a strong killer of *S. aureus* planktonic cells (EOP for >0.1).

The inhibitory biofilm growth activity of the different antibiotics was evaluated by monitoring the heat production for 72 h in the fresh medium according to Figure 1. Doxycycline displayed an inhibitory activity from 64 to >1024 μg/mL, levofloxacin and rifampin exhibited a similar inhibitory activity from 256 to >1024 μg/mL, whereas linezolid and clindamycin up to 1024 µg/mL showed no inhibitory activity across all six strains. The inhibitory activity of all the antibiotics obtained in the biofilm (MBIC) were from 32 up to 32,000 times higher than the planktonic form (MIC). All of the *S. aureus* biofilms exposed for 24 h to phage Sb-1 revealed a distinct heat-flow production reduction in each strain, but not complete inhibition compared to the growth control.

### 3.2. Synergistic Activity of the Phage–Antibiotic Combinations Inhibiting RRSA Biofilm Growth

**Figure 3:**
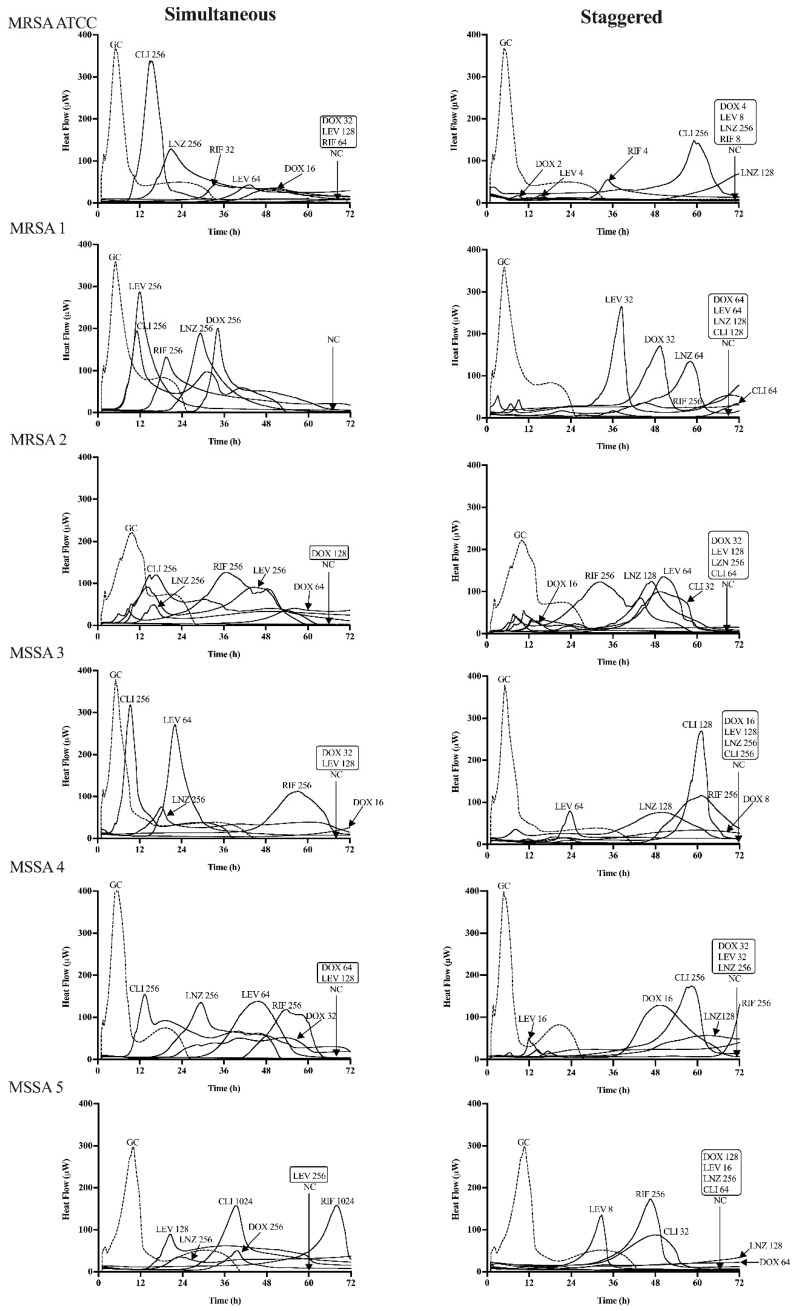
Microcalorimetry analysis of the reference strain MRSA ATCC 43300 and five rifampin-resistant *S. aureus* strains treated with phage and sub-inhibitory concentrations of antibiotic in a simultaneous (left column) or staggered (right column) manner. Each curve shows the heat produced by viable bacteria presented in the biofilm after 24 h of phage–antibiotic treatment. Numbers represent concentrations of antibiotic (in μg/mL) of doxycycline (DOX), levofloxacin (LEV), linezolid (LNZ), clindamycin (CLI), and rifampin (RIF). Circled values represent the MBIC, defined as the lowest antimicrobial concentration leading to the biofilm metabolism inhibition in the absence of antimicrobials for 72 h. GC, growth control; NC, negative control. Data of a representative experiment are reported.

The inhibitory biofilm growth activity of the phage–antibiotic combinations was evaluated in simultaneous and staggered exposure across all strains by IMC. The heat-flow production is described in Figure 3 for all of strains. Table 2 and Table S1 summarize the results of the MBIC obtained for simultaneous and staggered exposure of the phage–antibiotic combinations and the corresponding FBIC value.

The inhibitory biofilm growth activity of simultaneous application revealed no synergistic activity (Figure 3, Table 2 and Table S1), except the phage/rifampin combination in the MRSA ATCC 43300 strain. In contrast, when the reference strain MRSA ATCC and five RRSA biofilms were pre-treated with phage Sb-1 for 24 h, following exposure to sub-inhibitory concentrations of antibiotics, a strong reduction in the concentration of antibiotics was observed for the inhibition of the biofilm growth (Figure 3). Indeed, the best synergistic effect was achieved by the phage/doxycycline combination to inhibit all six *S. aureus* biofilms’ (100%) growth in a staggered manner, especially in the strains of MRSA 1 and MSSA 5 with the doxycycline-resistant profile. Similarly, the phage/linezolid combinations (100%) also showed a synergistic biofilm growth inhibition. However, the staggered phage/levofloxacin combination presented a synergistic inhibition improvement in five strains (83%), including the levofloxacin-resistant strains as well, whereas no synergistic activity appeared in the MSSA 3 (levofloxacin-susceptible) isolate. In addition, the phage/clindamycin combination had synergistic inhibitory activity only in four strains (67%). However, the phage/rifampin combination revealed no antimicrobial synergistic inhibitory improvement in a staggered manner for the five RRSA strains, except for the reference strain (Table S1), which was rifampin susceptible. Our results indicated the better biofilm inhibitory activity of the staggered application rather than the simultaneous one.

## 4. Discussion

The treatment of rifampin-resistant *S. aureus* implant-associated bone infections are challenging, causing a significant risk in patients and prone to treatment failure because of the limited therapeutic options. Thus, long-term oral antibiotic suppressive therapy may be the potential solution for maintenance of the foreign material when surgery is contraindicated. Due to the high penetration activity into osteoarticular tissue and good oral bioavailability, doxycycline, levofloxacin, linezolid, and clindamycin were recommended for the therapy of rifampin-resistant *Staphylococcal* PJIs [10,23], especially doxycycline because of its long half-life and acceptable tolerance [24,25]. Meanwhile, levofloxacin, linezolid, and clindamycin can be administered orally in combination treatment against *Staphylococcal* PJIs [26], markedly, linezolid and clindamycin have the ability to inhibit toxin production by inhibiting protein-synthesis, thus improving the clinical responses outcome [27]. However, the frequent long-term use of monotherapy may be potentially associated with several adverse events and proven not effective against chronic infection. For example, an increase in fluoroquinolone-resistant *S. aureus* has been reported [28,29], which is in agreement with our observations in the two MRSA strains.

Our study demonstrated that the MBIC value of a single antibiotic did not show superior inhibitory biofilm growth abilities, which is in line with other studies, defining that biofilm cells are highly tolerant to antibiotics compared to their planktonic counterparts [23,30]. Indeed, doxycycline and levofloxacin presented a better inhibitory biofilm growth ability compared to the bacteriostatic agents, such as linezolid and clindamycin. However, the choice of appropriate mono-antibiotic therapy may not be effective owing to unreachable clinical concentrations and developing antimicrobial resistance. Therefore, there is an urgent unmet requirement to develop new or alternative therapeutic strategies to control RRSA IABIs together with conventional antibiotics. Hypothetically, combination with bacteriophages may facilitate single antibiotics to enhance their activity, and this synergistic effect has been already observed in the context of eradication of biofilm infections [4,13]. However, to the best of our knowledge, our study is the first one proposing to use phage Sb-1 combination with current antibiotics to assess the optimal phage–antibiotics combination for inhibiting the RRSA biofilm growth activity.

Phage activity against biofilm cells has been reported to be in correlation with the anti-biofilm properties of the phage, rather than only on the phage’s lytic spectrum [31]. In our study, we also observed a high killing effect against planktonic cells of the tested strains (EOP, ranging from 0.5 to 0.9), but no biofilm eradication with the phage Sb-1 alone was observed, possibly due to the setting up of an equilibrium between the virus and host, which might be prevented with the augmentation of antibiotics [32].

Our investigation revealed that staggered administration significantly enhanced the inhibitory biofilm activity load on the glass beads surface compared with simultaneous exposure, corroborating previous reports [33,34]. It is hypothesized that pre-treatment with phages facilitates self-multiplication to reach a higher concentration for killing the sessile cells and disrupt the matrix of the biofilm, enabling the antibiotic to penetrate the deep layer of the biofilm by not decreasing therapeutic levels [34]. Antibiotics interfere with aspects of bacterial physiology that can facilitate phage antibacterial activities, which is exemplified by interference with bacterial ribosome functioning [35]. Unexpectedly, it is also noteworthy that in our study the greatest antibiofilm efficacy was observed in staggered administration of phage Sb-1 with both bactericidal and bacteriostatic antibiotics. Doxycycline and levofloxacin presented with a significantly low FBIC, followed by bacteriostatic drugs such as linezolid and clindamycin. Similarly, the ability to attack sessile cells, employing linezolid and clindamycin with the lytic phages, were reported by other authors using in vivo models [36,37]. However, we showed that the enhanced effect of combining phages and antibiotics sometimes was dependent not only on the order of exposure, but also on the host strain, wherein different biofilm inhibitory effects were demonstrated between the tested strains administrated to analogous phage–antibiotic combinations (Table 2).

Interestingly, we observed the synergistic activity of the phage–doxycycline combination and phage–levofloxacin combination in the doxycycline-resistant and the levofloxacin-resistant RRSA strains. One of the plausible antibiotic-resistant mechanisms could be the activation of the efflux pump protection system in the cell wall encoded by genes, actively removing doxycycline and levofloxacin from inside the cells [38,39], which is associated with moderate resistance reflected in the low MIC value in the resistant strains. Therefore, the possible mechanism can be attributed to the lytic phage Sb-1 targeting receptors in the cell wall that belong to the multidrug efflux systems [40,41]. In contrast, the synergistic activity of the phage-rifampin combination was not observed in the rifampin-resistant *S. aureus* strains, possibly due to the high-level resistance to rifampin by the clinical strains or to the rpoB mutations that alter the rifampin binding site on RNA polymerase [20], leading to affinity reduction.

Our study has some limitations related to the small numbers of available *S. aureus* strains. Moreover, the interaction of phage–antibiotic combinations needs to be evaluated by a full antibiotic chequerboard analysis. We just tested the activity of antibiotics with a two-log reduction concentration lower than the MBIC of some single antibiotics. In addition, a fixed value of 1024 μg/mL was defined for the calculation of the MBIC_phage_/MBIC_alone_ ratios for samples with MBIC_alone_ > 1024. Thus, by this approach, some combinations that were reported as not being synergistic could occur and have a synergistic effect when testing higher MBIC values.

## 5. Conclusions

Based on the above explanation, our findings demonstrate that the utilization of phage Sb-1 with doxycycline, levofloxacin, linezolid, and clindamycin might be a potential candidate for inhibiting rifampin-resistant *S. aureus* biofilm growth in a staggered manner, thus paving the way for effective treatment of implant-associated bone infections. Moreover, we showed that a phage–doxycycline combination appears to be the most efficacious therapy at decreasing biofilm-embedded *S. aureus* compared to other combinations, in an achievable concentration. Thus, this works sheds novel insights into the clinical application of phage–antibiotic combinations for long-term therapy of IABIs with available oral antibiotics against rifampin-resistant *S. aureus* biofilm-associated infections.

## Figures and Tables

**Table 1:** MIC and MBIC values (µg/mL) for the antibiotics tested against rifampin-resistant *S. aureus* strains.

Strains	DOX		LEV		LNZ		CLI		RIF	
	MIC	MBIC	MIC	MBIC	MIC	MBIC	MIC	MBIC	MIC	MBIC
MRSA ATCC	0.5	64	0.25	256	2	>1024	8 (R)	>1024	0.008	256
MRSA 1	16 (R)	>1024	4 (R)	>1024	2	>1024	0.25	>1024	1 (R)	>1024
MRSA 2	0.5	256	8 (R)	>1024	2	>1024	0.125	>1024	32 (R)	>1024
MSSA 3	0.125	64	0.125	256	1	>1024	0.25	>1024	32 (R)	>1024
MSSA 4	0.25	128	0.125	256	1	>1024	0.25	>1024	32 (R)	>1024
MSSA 5	16 (R)	>1024	0.5	512	1	>1024	0.125	>1024	1 (R)	>1024

Abbreviation: DOX, doxycycline; LEV, levofloxacin; LNZ, linezolid; CLI, clindamycin; RIF, Rifampin. R: resistance against the antibiotic according to EUCAST.

**Table 2:** Synergistic inhibitory effects of simultaneous (SIM) and staggered (STA) exposure of phage–antibiotic combinations.

Strains	DOX (SIM)		LEV (SIM)		LNZ (SIM)		CLI (SIM)	
	MBIC	FBIC	MBIC	FBIC	MBIC	FBIC	MBIC	FBIC
MRSA ATCC	32	0.5 (NS)	128	0.5 (NS)	>256	>0.25 * (NS)	>256	>0.25 * (NS)
MRSA 1	>256	>0.25 * (NS)	>256	>0.25 * (NS)	>256	>0.25 * (NS)	>256	>0.25 * (NS)
MRSA 2	128	0.5 (NS)	>256	>0.25 * (NS)	>256	>0.25 * (NS)	>256	>0.25 * (NS)
MSSA 3	32	0.5 (NS)	128	0.5 (NS)	>256	>0.25 * (NS)	>256	>0.25 * (NS)
MSSA 4	64	0.5 (NS)	128	0.5 (NS)	>256	>0.25 * (NS)	>256	>0.25 * (NS)
MSSA 5	>256	>0.25 * (NS)	256	0.5 (NS)	>256	>0.25 * (NS)	>256	>0.25 * (NS)
**Strains**	**DOX (STA)**		**LEV (STA)**		**LNZ (STA)**		**CLI (STA)**	
	**MBIC**	**FBIC**	**MBIC**	**FBIC**	**MBIC**	**FBIC**	**MBIC**	**FBIC**
MRSA ATCC	4	0.06 (S)	8	0.03 (S)	256	0.25 (S)	>256	>0.25 * (NS)
MRSA 1	64	0.06 * (S)	64	0.06 * (S)	128	0.13 * (S)	128	0.13 * (S)
MRSA 2	32	0.13 (S)	128	0.13 * (S)	256	0.25 * (S)	64	0.06 * (S)
MSSA 3	16	0.25 (S)	128	0.5 (NS)	256	0.25 * (S)	256	0.25 * (S)
MSSA 4	32	0.25 (S)	32	0.13 (S)	256	0.25 * (S)	>256	>0.25 * (NS)
MSSA 5	128	0.13 * (S)	16	0.03 (S)	256	0.25 * (S)	64	0.06 * (S)

Abbreviation: DOX, doxycycline; LEV, levofloxacin; LNZ, linezolid; CLI, clindamycin. MBIC, minimum biofilm inhibitory concentration (values are expressed in μg/mL). FBIC, fractional biofilm inhibitory concentration; in brackets is shown the interpretation, S: Synergism; NS: No-Synergism. * MBIC of the single antibiotic was considered equal to 1024 μg/mL for the MBICphage/MBICalone ratio calculations.

## Supplementary Materials

The following are available online at https://www.mdpi.com/2079-6382/9/11/749/s1, Table S1: Synergistic inhibitory effects of simultaneous and staggered exposure of phage-rifampin combination.
