# Supplementary material for: Adjunctive Use of Phage Sb-1 in Antibiotics Enhances Inhibitory Biofilm Growth Activity versus Rifampin-Resistant Staphylococcus aureus Strains
Source: Antibiotics (Basel). 2020 Oct 29;9(11):749. doi: 10.3390/antibiotics9110749 (PMC7692760; doi:10.3390/antibiotics9110749)
Supplement: Supplementary file 1 [file antibiotics-09-00749-s001.pdf]

antibiotics

Supplementary Materials

Adjunctive Use of Phage Sb-1 to Antibiotics Enhances Inhibitory Biofilm Growth Activity versus Rifampin-resistant *Staphylococcus aureus* strains

Lei Wang [1,2,†], Tamta Tkhilaishvili [1,2,†] and Andrej Trampuz [1,2,*]

1 Center for Musculoskeletal Surgery, Charité—Universitätsmedizin Berlin, Corporate Member of Freie Universität Berlin, Humboldt-Universität zu Berlin and Berlin Institute of Health, 13353 Berlin, Germany; lei.wang@charite.de (L.W.); tamta.tkhilaishvili@charite.de (T.T.)
2 Berlin-Brandenburg Center for Regenerative Therapies, Charité—Universitätsmedizin Berlin, 13553 Berlin, Germany
* Correspondence: andrej.trampuz@charite.de; Tel.: +49-30-450-615-073
† These authors contributed equally to this work.

Table S1. Synergistic inhibitory effects of simultaneous and staggered exposure of phage-rifampin combination.

**Strains**	**RIF (SIM)**	
	**MBIC**	**FBIC**
MRSA ATCC	64	0.25 (S)
MRSA 1	> 256	> 0.25* (NS)
MRSA 2	> 256	> 0.25* (NS)
MSSA 3	> 256	> 0.25* (NS)
MSSA 4	> 256	> 0.25* (NS)
MSSA 5	> 256	> 0.25* (NS)
Strains	RIF (STA)	
	MBIC	FBIC
MRSA ATCC	8	0.03 (S)
MRSA 1	> 256	> 0.25* (NS)
MRSA 2	> 256	> 0.25* (NS)
MSSA 3	> 256	> 0.25* (NS)
MSSA 4	> 256	> 0.25* (NS)
MSSA 5	>256	> 0.25* (NS)

Abbreviation: DOX, doxycycline; LEV, levofloxacin; LNZ, linezolid; CLI, clindamycin. MBIC, minimum biofilm inhibitory concentration (values are expressed in μg/mL). FBIC, fractional biofilm inhibitory concentration; in brackets is shown the interpretation, S: Synergism; NS: No-Synergism. *MBIC of the single antibiotic was considered equal to 1024 μg/mL for MBICphage/MBICalone ratio calculations.
